# Supplementary material for: Are carbon emissions trading and green financial instruments synergistic? -Comprehensive quantitative research based on content analysis
Source: PLoS One. 2024 Mar 7;19(3):e0298601. doi: 10.1371/journal.pone.0298601 (PMC10919601; doi:10.1371/journal.pone.0298601)
Supplement: S1 Data — (ZIP) [file pone.0298601.s001.zip › Data/地级市Z_F工作报告.zip!/╕ú╜¿/╕ú╓▌╩╨/└┤╫╘╣1⁄2╓┌║┼í╛┬φ┐╦╩2╛▌═°í┐_2011.docx]

各位代表：

　　现在，我代表福州市人民政府向大会作政府工作报告，请各位代表审议，并请市政协各位委员和其他列席人员提出意见。

　　一、2011年暨市十三届政府工作回顾

　　刚刚过去的2011年，在省委、省政府和市委的正确领导下，我市各级政府紧紧围绕科学发展这一主题，牢牢把握加快转变、跨越发展这一主线，认真实施《海峡西岸经济区发展规划》，大干“十二五”开局之年，较好地完成了市十三届人大六次会议确定的任务。预计全市生产总值3705亿元，增长13%；财政总收入506.01亿元，增长25.7%，其中，地方财政收入320.04亿元，增长29.1%；全社会固定资产投资2800亿元，增长27%；出口总额237亿美元，增长45.3%；按验资口径实际利用外资12.77亿美元，增长7.8%；社会消费品零售总额1866亿元，增长18%；城镇居民人均可支配收入26040元，增长14.6%；农民人均纯收入10080元，增长18%；城镇登记失业率2.6%；人口自然增长率5.23‰；省下达的节能减排任务预计可以完成。在过去的一年，福州荣膺全国文明城市称号，全市人民经过多年来不懈努力终于圆梦，福州发展迈上了一个崭新的台阶。一年来，我们的主要工作及成效是：

　　（一）着力转变发展方式，产业转型升级加快推进。坚持抓龙头、铸链条，有88项重点工业项目竣工投产，97项重大工业项目动工建设。规模以上工业总产值5500亿元，增长16.1%。高新技术产业工业总产值1869亿元，增长18%。高新技术产业标准化示范区试点工作通过国家验收。制定出台鼓励发展总部经济、会展经济、研发中心、创意产业等一系列政策措施，服务业发展水平进一步提升，福州成为首批创建国家电子商务示范城市之一。组建成立福州农村商业银行，创建海峡沪深指数，设立海西商品交易所，金融业加快发展。温泉游、闽江游、文化游持续升温，全市接待游客2696.75万人次。文化产业蓬勃兴起，年增加值177.8亿元。成功举办中国国际医疗器械博览会等222场全国性、区域性展会。

　　（二）着力强化项目带动，经济发展后劲明显增强。实施“五大战役”项目485项，完成投资1123亿元，为年度计划的116.9%。实施市级重点项目535项，完成投资1148亿元，为年度计划的111%，建成或基本建成100个项目，新开工建设160个项目。大力加强“三维”对接，成功举办与中央企业项目合作洽谈暨签约仪式、民营企业产业项目洽谈会、赴港澳经贸洽谈活动，签约对接央企项目21项、总投资2256亿元，民企项目64项、总投资1216亿元，外资项目75项、总投资98.85亿美元。

　　（三）着力强农惠农富农，“三农”基础地位不断夯实。推进农业规模化、设施化、标准化生产，粮食生产保持稳定，农林牧渔业总产值565.3亿元，增长4%，新增设施大棚1.23万亩，209家农业产业化龙头企业年产值突破400亿元。福州被国际茶叶委员会授予“世界茉莉花茶发源地”称号。加强耕地保护，大力推进水利改革发展，实施标准农田改造2900亩，土地整理开发复垦2.03万亩，标准化养殖池塘改造7256亩，除险加固水库16座。全面完成省级试点小城镇、市级示范性小城镇总体规划编制，青口、荆溪、龙田、江田等小城镇启动区动工建设。启动第三轮新农村“双百工程”建设，新建和改造农村公路389.6公里，解决19.41万农村人口饮水安全问题，新建户用沼气池2300口，改造卫生户厕1.44万户，农村生产生活条件持续改善。实施第三轮整村推进扶贫开发，完成“造福工程”搬迁4500人。

　　（四）着力改善宜居品质，城市功能品位持续提升。编制完成新一轮城市总体规划，启动福州大都市区规划编制，深化马尾新城分区规划和南台岛、晋安等重点建设区域城市设计。加快中心城市东扩南进、沿江向海发展，海峡金融商务区等城市组团建设扎实推进，三环快速路主线通车，地铁1号线站点建设全面启动。新建和改扩建城市道路23条，新增更新公交车785辆，新辟公交线路40条，新增停车泊位3070个。认真实施“四绿”工程建设，超额完成省下达的88.1万亩造林任务，新增100万平方米城市公共绿地。全力打好内河整治攻坚仗，中心城区75条内河除光明港外基本完成清淤，安泰河、白马河、晋安河综合整治初见成效。深入开展市容环境卫生、交通秩序、违法建设等20项专项整治，全面清理城区户外广告，基本完成城区主干道架空缆线下地。实施节能重点项目70个、减排重点项目184个，实现重点污染源自动监控全覆盖，创建40个省级以上生态乡镇、700个市级以上生态村，以较好的成绩通过了创卫复查和创模省级复核。

　　（五）着力推进先行先试，重点领域改革全面突破。落实省里赋予的在城市总体规划管理和财政体制方面先行先试的政策，统筹推进福清、长乐、闽侯、连江等周边县(市)融入中心城市发展格局。深化行政审批制度改革，下放10类重点项目审批事项，赋予县（市）区更大发展自主权。强化国资运营监管，市属投融资平台、产业集团和专业公司加快发展。全面落实促进民营经济和小型微型企业发展的政策措施。积极拓展直接融资渠道，BT投融资建设模式得到有效应用，新榕城建等融资平台成功申报企业债券29亿元，2家企业在境内外上市。深化社会事业领域改革，完成医药卫生体制改革三年任务，义务教育“小片区”管理改革试点取得较好成效。集体林权制度配套改革持续深化。政府机构改革、事业单位人事制度改革扎实推进。

　　（六）着力提升对外开放，交流合作空间有效拓展。对外贸易在外需不足的情况下保持较快增长，进出口总额343亿美元，增长39.4%，机电、高新技术产品出口增长22%，福州成为全国首批船舶出口基地之一。招商引资实效进一步提高，新批千万美元以上外（台）资项目57项。福州保税港区（一期）通过国家验收。积极落实海峡两岸经济合作框架协议，榕台经贸合作和空中直航、海上直航不断拓展，福清台湾农民创业园获得国家批准，马尾船政文化园区获批成为海峡两岸交流基地。成功举办第十三届海交会以及海峡两岸合唱节、海峡版权(创意)产业精品博览交易会、海峡（福州）渔业周暨渔博会、两岸城市青少年创意族谱联展等对台特色交流活动。积极支持和融入平潭开放开发，与平潭综合实验区签订合作框架协议。主动推进福莆宁同城化发展。全面完成对口支援三明灾后重建任务，积极推进新一轮援疆、援藏工作。成功举办纪念福州与日本那霸缔结友好城市30周年等友城交流活动。

　　 （七）着力完善公共服务，各项社会事业协调发展。加快发展各级各类教育，新建和改扩建23所中小学，完成522所义务教育学校标准化建设，基本完成中小学校安工程三年建设任务，实现每个乡镇（街道）至少有一所公办幼儿园，校园文化建设、规范办学行为等工作得到中央、省充分肯定。认真实施基本和重大公共卫生服务项目，进一步提高城镇居民医保、新农合补助标准和基本公共卫生服务人均经费标准，动工建设福州儿童医院等4家医院病房楼，在全省率先建设社区医疗康复体系。加强历史文化名城保护，完成第三次文物普查任务，“三坊七巷”社区博物馆成为全国首家生态（社区）博物馆。积极推进文化惠民工程，提前一年实现所有行政村都建有一家农家书屋。文艺精品创作成效喜人，闽剧《王茂生进酒》入选国家舞台艺术精品工程。成功申办第八届全国城运会，参加第七届全国城运会取得优异成绩，福州成为全国全民健身示范城市试点单位。低生育水平保持稳定，人口与计划生育优质服务进一步加强。民族团结进步创建活动深入开展，宗教事务依法管理更加规范。

　　（八）着力多办惠民实事，人民生活水平稳步提高。全市各级财政用于民生支出239.65亿元，占公共财政预算支出的65.6%，市本级财政超收的82.3%用于改善民生。年初确定的20件125项为民办实事项目件件有落实。城乡就业保持稳定，城镇新增就业15.32万人，转移农业富余劳动力5.88万人。最低工资标准和企业离退休人员养老金、村（社区）“两委”成员补贴、企业军转人员生活补贴、公益性岗位临时人员工资进一步提高。城镇居民养老保险试点全面展开，新型农村社会养老保险制度实现全覆盖，城乡低保、农村“五保”供养标准进一步提高。社区居家养老服务工作获得“中国城市管理进步奖”。实施663万平方米危旧房（棚屋区）改造，超额完成省下达的保障性安居工程开工建设任务。在全国率先实施蔬菜、猪肉价格协商制度，实行物价上涨与社会救助和保障标准挂钩联动，建设51家平价店，开通“菜篮子”社区平价直通车，较好地平抑市场物价，居民消费价格总水平上涨4.7%，低于全省平均水平。组织开展食品安全大整治，在全国率先将食品安全纳入综治考评，全市食品安全检测全部达标。加强和创新社会管理，流动人口、特殊人群、社会组织、信息网络等服务管理机制不断完善。深化“平安福州”建设，安全生产事故下降20.2%，第三季度人民群众对社会治安满意率达94.75%。积极解决信访积案和历史遗留的留用地补偿、“两权证”办理等问题，及时排查化解社会矛盾纠纷，有效维护省会中心城市和谐稳定。

　　（九）着力优化行政服务，政府自身建设切实加强。坚持依法行政，全面梳理行政职权，集中清理市政府规章、规范性文件，提请市人大常委会审议地方性法规草案2项，制定政府规章和规范性文件42件。组建成立市行政服务中心，90%以上的行政审批、公共服务事项实行集中办理，做到一个窗口受理、“一条龙”服务、“一站式”办结。建立深入企业常态化服务机制，积极开展“服务企业周”等活动。加强机关效能建设，严格绩效管理，坚决治庸治懒，实施效能告诫133人次。健全完善惩治和预防腐败体系，加强行政监察和审计监督，切实治理工程建设领域突出问题及“小金库”、公务用车等问题，廉政建设和反腐败工作进一步加强。构建完善行政权力阳光运行平台，开通网上办事公开系统。认真执行人大及其常委会决定决议，自觉接受人大及其常委会的法律监督、工作监督和政协的民主监督，积极支持人大、政协开展检查、视察、调研等活动，广泛听取各民主党派、工商联、人民团体和社会各界人士意见建议，办复517件省、市人大代表建议、批评、意见和536件省、市政协提案，满意率分别为94%和99.4%。　2011年各项任务的完成，为市十三届政府的工作划上了圆满的句号。回顾过去的五年，勤劳智慧的福州人民，积极抢抓海西建设机遇，有效应对国际金融危机等挑战，用激情与拼搏，谱写了福州科学发展、跨越发展的辉煌篇章。

　　 通过五年来的努力，综合实力显著增强。全市生产总值年均增长13.9%。财政总收入突破500亿元，地方财政收入突破300亿元，规模以上工业总产值突破5500亿元，全社会固定资产投资突破2800亿元，社会消费品零售总额突破1800亿元，出口总额突破230亿美元，均比2006年翻了一番以上。福州相继获得国家知识产权工作示范城市、全国未成年人思想道德建设工作先进城市、国家创新型试点城市、全国绿化模范城市、全国流通领域现代物流示范城市、中国温泉之都、全国文明城市等称号，并蝉联全国科技进步先进市、全国双拥模范城等称号。

　　通过五年来的努力，经济结构不断优化。全市三次产业比例已达8.9∶45.8∶45.3。农业向产业化、特色化、现代化方向发展，形成水产、果蔬、食用菌、茶叶、花卉、竹木等特色优势产业。工业加速向南北“两翼”集聚，电子信息、冶金建材、石油化工、生物医药、新材料及能源等重点产业集群不断壮大，形成机械装备、纺织服装、轻工食品等3个千亿产业集群和4家百亿企业。商贸、物流、金融、旅游、文化创意、服务外包等服务业加快发展，城乡消费市场繁荣活跃。自主创新体系不断完善，经认定的高新技术企业达342家，研发投入占地区生产总值的比重由1.32%提高到1.81%，累计创建国家级创新型（试点）企业5家、国家级企业技术中心3家、行业技术创新中心34家、院士（专家）工作站62家，中科院海西研究院落地建设。县域经济实力持续增强，占全市经济总量的比重达52%。

　　通过五年来的努力，城乡面貌深刻变化。城市建成区面积从177.19平方公里扩展到250平方公里，城镇化率从55.5%提高到62.9%。建成温福、福厦高速铁路福州段，高速公路总里程从275公里增加到427公里，长乐国际机场年旅客吞吐量从379万人次增长到725万人次，福州港成为国家主要港口之一。城市道路总长从275公里增加到870公里，公交出行率从17.3%提高到22.1%，水、电、气、通讯等承载服务能力与城市防灾减灾能力有效增强，市容环境卫生和园林绿化水平切实提升。城市环境综合整治定量考核成绩持续位居全国前列，在中国环境规划院公布的宜居城市监测评价结果中福州位居首位。新农村建设扎实推进，基本实现村村通水泥路、客运班车和广播电视，“万村千乡”市场工程建设基本覆盖所有乡镇，累计完成“造福工程”搬迁2.46万人，帮扶2.38万农村人口脱贫。

　　 通过五年来的努力，改革开放持续深化。市属国有企业改革和公用事业企业改制基本完成，国有资产监管体系全面建立。民营经济持续健康发展。公共资源配置、公共资产交易、公共产品生产等领域的市场运行机制加快完善，土地使用权“招拍挂”出让、工程建设项目招投标、政府采购、产权交易等制度全面实施。农村综合改革不断深化，农村工作机制持续创新。投融资、公共财政、政府机构、行政审批、社会管理及科技、教育、文化、医药卫生等领域改革取得良好成效。对外开放水平进一步提高，按验资口径累计实际利用外（台）资51.69亿美元，在榕投资设点的世界500强企业达87家。榕台交流合作不断取得先行先试的突破，率先实施中央惠台政策，率先开通两岸海上直航，率先签订两岸城市合作协议，率先推动两岸双向投资，率先出台台湾居民申办个体工商户、台商子女入学等政策措施。榕港榕澳合作、海内外友城合作、闽浙赣皖经济协作、闽东北五市区域协作、山海协作、对口支援以及侨务、外事、异地商会等工作扎实推进。

　　通过五年来的努力，社会事业长足进步。教育均衡化、信息化、现代化水平不断提升，在全省率先实现农村地区“双高普九”，鼓楼区获得全国推进义务教育均衡发展工作先进地区称号，马尾区、长乐市率先实行高中阶段免费教育，农民工子女入学、数字青少年宫建设、教育督导、乡土文化传承等工作走在全国前列。覆盖城乡的医疗卫生服务体系、疾病预防控制体系和突发公共卫生事件应急体系基本建立，社区卫生服务中心、乡镇卫生院实现全覆盖，居民健康主要指标居全省前列。精心打造“三坊七巷”文化、船政文化、昙石山文化、寿山石文化等四大闽都文化品牌，“三坊七巷”入选中国十大历史文化名街，寿山石雕等13项非物质文化遗产入选国家保护名录，3个村（镇）入选国家历史文化名村（镇）。10项文艺精品获得全国性大奖，“激情广场大家唱”等群众文化活动形成品牌效应。成功举办全国特奥会等重大赛事，竞技体育屡创佳绩，全民健身活动蓬勃开展。人口计生、民族宗教、新闻出版、广播影视、哲学社会科学、科普、防震、地方志、档案、老龄、妇女儿童、残疾人等各项社会事业全面发展。

　　通过五年来的努力，民生福祉切实改善。全市各级财政累计用于民生支出718.37亿元，累计为城乡人民兴办实事项目91件351项。城镇居民人均可支配收入、农民人均纯收入分别比2006年增长83.3%和80.3%。累计实现城镇新增就业75.5万人次，转移农业富余劳动力33.9万人次。社会保险覆盖面不断扩大、标准持续提高，低收入家庭生活保障、廉租住房保障应保尽保。累计建成保障性住房8.4万套，改造危旧房（棚屋区）1706.97万平方米。“数字福州”建设扎实推进，便民呼叫中心12345系统、“中国福州”门户网站、政府信息公开、行政权力阳光运行平台建设等工作走在全国前列。社会救助、社会福利、社会优抚、社会慈善加快发展，各类扶贫济困活动广泛开展。“平安福州”建设取得良好成效。信访、人民调解、普法、法律援助、法律服务、社区矫正、青少年事务社工以及国防动员、双拥共建、民兵预备役、海防、人防、反走私等工作进一步加强。

　　各位代表，回顾过去五年的工作，我们深深体会到，做好福州的工作，必须始终坚持科学发展不动摇、不懈怠，突出加快发展，以等不起的紧迫感抢抓机遇，以慢不得的危机感跨越赶超，以坐不住的责任感干事创业；突出创新发展，不断在解放思想中开拓创新，在先行先试中率先突破；突出特色发展，充分发挥福州省会、侨台、海洋、文化、生态等比较优势，彰显福州个性、特色与魅力，凸显省会城市的龙头引领作用；突出统筹发展，坚持走全面协调可持续发展之路；突出和谐发展，切实让最广大人民群众更多地共享发展成果。

　　回首过去的五年，我们走过的道路并不平坦，取得的成绩来之不易，这是省委、省政府和市委正确领导的结果，是全市人民团结奋斗的结果，是社会各界大力支持的结果。在此，我代表市人民政府，向为福州建设发展付出辛勤劳动的全市人民，向给予政府工作有力支持与有效监督的人大代表和政协委员、各民主党派、工商联、各人民团体、无党派人士、离退休老同志及社会各界人士，向为福州建设发展作出积极贡献的中央、省直机关企事业单位和驻榕部队、武警官兵、公安政法干警，向关心支持福州发展的香港特别行政区同胞、澳门特别行政区同胞、台湾同胞、海外侨胞、国际友人，表示衷心的感谢！

　　 在看到成绩的同时，我们也清醒地认识到，当前福州经济社会发展仍面临不少困难和问题：一是思想观念、体制机制某些方面还不适应科学发展、跨越发展的要求；二是经济综合实力与核心竞争力不够强，创新创业领军人才较为缺乏；三是城市规划建设管理水平还需要进一步提升，交通拥堵、违法建设等问题还比较多；四是城乡之间、县（市）区之间发展不够平衡；五是住房、物价、食品安全等民生问题需要继续认真解决，部分群众生活还比较困难；六是社会管理还需要加强，治安防控体系还不健全，安全生产事故、群体性事件仍时有发生；七是政府效能有待提高，不正之风、消极腐败现象仍然存在。我们要正视这些问题，采取更加积极有效的措施，认真加以解决。　　二、新一届政府努力方向和2012年工作意见

　　各位代表，根据市第十次党代会部署，今后五年我市经济社会发展的总体要求是：高举中国特色社会主义伟大旗帜，以邓小平理论和“三个代表”重要思想为指导，深入贯彻落实科学发展观，牢牢把握海西建设重大机遇，进一步敢为、能为、有为，坚持以科学发展为主题，以加快转变、跨越发展为主线，以改革开放为动力，“三化”并举、“三群”联动、“三维”对接，在更高起点上加快富民强市、和谐宜居进程，建设开放、文明、和谐、幸福的新福州。今后五年，新一届政府的主要任务：一是全面完成“十二五”发展规划，力争地区生产总值比2011年翻一番以上，人均地区生产总值率先赶超东部地区平均水平，地方财政收入翻番；二是认真实施《海峡西岸经济区发展规划》，加快构建福州大都市区，凸显省会城市的龙头引领作用；三是争取率先建成惠及全市人民的小康社会。为此，新一届政府任重道远，应在以下方面作出不懈努力：

　　——努力在解放思想上实现新突破。坚持以解放思想为先导，敢于突破常规、敢于先行先试、敢于担当责任，坚决破除自满、畏难、守旧的思想障碍，增强干事创业的志气、勇气和锐气。牢固树立和增强“大福州”观念，进一步站位全局、开阔视野，营造比学赶超的良好发展氛围。全面深化收入分配、国有资产、投融资、公共财政、行政管理、社会管理、公共服务等重点领域改革，推进城乡统筹综合配套改革试验，让发展活力竞相迸发、生产要素集聚涌流。

　　 ——努力在产业提升上实现新突破。增强传统产业竞争优势、提高服务业比重、培育壮大战略性新兴产业，打造海峡西岸先进制造业基地和商贸物流中心、自然文化旅游中心、金融服务中心、信息研发中心、服务外包基地及海洋经济强市，争取形成电子信息、机械装备、纺织服装、石油化工、轻工食品、冶金建材等6个千亿产业集群，培育15家以上百亿企业。构建完善以企业为主体、市场为导向、产学研相结合的区域技术创新体系，大力推进原始创新、集成创新和引进消化吸收再创新，争创国家创新型城市。

　　 ——努力在对外开放上实现新突破。充分利用国内国外两个市场、两种资源，推进全方位、高水平的大开放，增创以技术、品牌、质量、服务为核心竞争力的开放新优势。建设两岸交流合作先行城市，打造两岸经贸合作的紧密区域、文化交流的重要基地和直接往来的综合枢纽。大力实施“人才强市”战略，推进闽都人才集聚工程，打造海西大都市区人才高地。把环境作为对外开放第一竞争力，按国际惯例办事，按国际水平服务，使福州真正成为投资发展的“福地”和“沃土”。

　　 ——努力在统筹发展上实现新突破。更加积极地统筹城乡一体发展，构筑“一核引领、两翼崛起、三带并进”的福州大都市区新格局，建设富裕、民主、文明、和谐的社会主义新农村。更加积极地统筹区域发展，促进与平潭综合实验区的互动联动发展和福莆宁同城化发展，提升对口支援、山海协作、区域协作水平。更加积极地统筹经济社会协调发展，推动社会事业全面进步、文化繁荣发展，争取文化、教育、卫生、体育发展水平进入全国省会城市前列，确保成功举办第八届全国城运会。更加积极地统筹人与自然和谐发展，推进资源节约型和环境友好型社会建设，争创国家生态市。

　　 ——努力在改善民生上实现新突破。积极推动城乡居民收入与经济发展同步增长，力争实现倍增。建立健全统筹城乡的社会保障体系和公共就业服务体系，切实保障学有所教、劳有所得、病有所医、老有所养、住有所居。加强和创新社会管理，不断提高基本公共服务均等化程度，促进社会公平正义，深化“平安福州”建设，进一步打造人民满意的幸福城市。各位代表，2012年是新一届政府的开局之年。当前世界经济复苏的不稳定性、不确定性上升，我们面临的形势更加复杂严峻。做好2012年政府工作，必须牢牢把握稳中求进的总基调，以“稳”为基础，“进”为关键，在“稳”的基础上更多地着力在“进”，突出加快开放型经济发展，进一步稳增长、控物价、调结构、惠民生、抓改革、促和谐。初步安排2012年全市经济社会发展主要预期目标是：地区生产总值增长12%；地方财政收入增长18%；全社会固定资产投资增长18%；出口总额增长12%；按验资口径实际利用外资13亿美元；社会消费品零售总额增长15%；居民消费价格总水平涨幅控制在4%左右；城镇居民人均可支配收入增长12%，农民人均纯收入增长12%；城镇登记失业率控制在3.5%以内；人口自然增长率控制在7.6‰以内；确保完成省下达的节能减排任务。为此，重点应抓好以下工作：

　　（一）进一步转变发展方式，保持经济持续平稳较快发展。

　　 强化内需拉动。坚持不懈打好“五大战役”，大力推进重点项目建设。初步安排“五大战役”项目520项，总投资6667亿元，年度计划投资968亿元；市级重点项目580项，总投资8700亿元，年度计划投资1200亿元。积极拓宽消费领域，促进居民文化、旅游、健身、养老、家政等服务消费，鼓励发展电子商务、信贷消费等新型消费方式。积极申办全国性、区域性展会，大力拓展会展消费。加强农村流通市场体系建设，继续实施家电下乡和“新网工程”建设，积极开拓农村消费市场。繁荣社区商业，实施“便利消费进社区，便民服务进家庭”工程。

　　 强化创新驱动和品牌带动。积极推进国家创新型试点城市建设，加快建设中科院海西研究院，争取新创建一批国家级、省级创新型企业与企业技术中心。加强科技成果转化运用，促进资金、技术、人才、市场的有效对接，发展行业技术创新中心和软件设计、集成电路、工业设计、动漫娱乐、信息传媒等公共创新服务平台，扩建金山科技企业孵化器。认真实施制造业信息化工程，支持企业加快技术改造和产品升级。提升知识产权创造、运用、保护和管理水平。大力推进企业品牌建设工程，培育、引进、扶持和保护一批品牌商标、名牌产品。落实和扩大甲供甲控政策，积极支持榕货名优产品拓展海内外市场。

　　 强化对企业及时有效帮扶。健全深入企业常态化服务机制，落实和完善促进企业发展的各项政策，“一企一策”加大对重点企业的帮扶力度。深化政银企合作，建立重点企业资金链保障机制，完善中小企业融资担保体系。拓展债权融资、风险投资等直接融资渠道，支持有条件的企业在境内外上市。加强对企业的用工服务与人才指导，着力帮助企业解决用工困难。支持企业参加重点展会。对小型微型企业免征部分行政事业性收费。从严控制并切实减少涉企的各种检查、评比、表彰、达标、庆典、论坛、研讨会、培训、评价评估等活动。

　　（二）进一步推进产业转型升级，做大做强实体经济。

　　 加快发展现代农业。全面落实强农惠农富农政策，毫不放松地抓好粮食生产，发展壮大水产、果蔬、食用菌、茶叶、花卉、竹木等特色优势产业，保障“米袋子”、“菜篮子”安全。鼓励农业规模化、标准化生产和产业化经营，大力发展高效设施农业、绿色生态农业、休闲观光农业和农产品精深加工业、流通业，支持农业品牌创建，推进福清龙田全国农产品加工示范基地等现代农业基地建设。加强林业“五大工程”、“五大基地”建设。积极促进“农超对接”和“产加销一条龙、农工商一体化”，培育壮大农业产业化龙头企业、农民专业合作组织。完善农业科技推广体系，加强农业“五新”推广和农技服务。切实防控动植物疫病，严格农产品质量监管，积极推广畜牧、水产健康生态养殖。加强农村土地整理开发复垦，落实补充耕地任务。

　　 加快发展先进制造业和高新技术产业。认真抓好蓝星化工、中国软包装、中化工程己内酰胺、宝钢德盛不锈钢、兆元光电等一批重大产业项目建设，争取引进落地中航国际通用飞机制造、神华煤电一体化等一批产业龙头项目，力争新培育3家以上百亿企业和1～2个千亿产业集群。大力推动产业向园区集中，提升融侨、元洪、江阴、滨海、青口等现有工业集中区，加快发展可门港工业区、长乐不锈钢深加工产业园、连江铝深加工园、福清光电科技园及洪宽机电园、长乐空港工业区、闽侯经济开发区、罗源湾开发区、闽清白金工业园等一批新兴特色产业园区。鼓励企业组建战略联盟。培育壮大新一代信息技术、生物与新医药、节能环保、光电、软件、物联网等高新技术产业，加快建设海西高新技术产业园、生物医药和机电产业园、福兴智能化产业园区，推动普天新一代信息产业研发和生产基地、联通东南区域云计算产业园、移动福建数据（云计算）中心等大项目落地，力争新增50家以上高新技术企业。

　　加快发展现代服务业。积极培育“两江四岸”高端服务业产业带，打造海峡金融商务区、闽江北岸中央商务区等现代服务业集聚区，加快建设泰禾城市广场、苏宁广场等一批城市综合体，争取引进落地中石油、中石化福建区域总部及华润城市综合体、中金国际交易中心等项目。积极推进海峡钢贸城、医药城、建材市场和中储粮长乐直属库等项目，加快建设物流分拨中心、配送中心，发展第三方物流企业。推进鼓楼区国家服务业综合改革试点和洪山先进技术服务产业园、台江区海峡电子商务产业基地（二期）建设，培育壮大总部经济、楼宇经济、会展经济。做大做强福建海峡银行、福州农村商业银行，鼓励在榕金融机构发展壮大，广泛吸引金融机构落户福州，支持发展产权交易中心、债券市场等金融平台和小额贷款公司、村镇银行等金融组织，加快建设海西现代金融中心。发展温泉游、闽江游、文化游、生态游、乡村游，推进三江口、贵安、桂湖、琅岐、青云山、东壁岛、鼓岭等一批高端旅游综合体建设和旅游片区开发，建成温泉博物馆，争取“三坊七巷”创建5A级旅游景区，加快打造海西重要旅游目的地。推进游艇码头建设，培育发展游艇产业。大力发展服务外包产业，争取进入中国服务外包基地城市行列。

　　 加快发展海洋经济。紧紧抓住建设海峡蓝色经济试验区的机遇，全面推进“海上福州”建设。认真实施“以港兴市”战略，加强福州港资源整合、泊位建设、航道疏浚、航线开辟，积极打造国际航运枢纽港。加快福州保税港区建设发展，充分发挥其政策功能优势及作用。大力推进“两翼”港区基础设施建设，发展壮大临港工业、港口物流、船舶修造、海洋运输、滨海旅游、海洋生物医药等“蓝色产业”，加速构建罗源湾、闽江口、福清湾、兴化湾北岸等海洋经济集聚区。加快海峡西岸（连江）水产品加工基地、海西远洋渔业产业园建设，扶持壮大远洋渔业、水产加工业，有序拓展湾外养殖。实施“碧海银滩”工程，科学合理开发海域、岸线和海岛资源。

　　（三）进一步加快构建福州大都市区，统筹推进城乡一体发展。

　　全面构建大都市区发展新格局。高起点、高标准、高水平地加快编制福州大都市区发展规划。全力推动马尾新城和南台岛、晋安等区域成片开发建设。统筹推进福清、长乐、闽侯、连江等周边县（市）同城化发展，加快拓展中心市区。加大扶持力度，积极把闽清、罗源、永泰培育成为大都市特色功能承载区。加快推动福莆宁交通互联互通、产业分工协作、公共服务互动对接，促进福莆宁融合发展、连片繁荣。加强闽东北五市区域协作，推动福州—宁德—南平—鹰潭—上饶发展轴联动发展，壮大海峡西岸东北翼增长极。

　　大力建设马尾新城。把马尾新城作为中心城市东扩南进、沿江向海的主攻方向和新区拓展的重要载体，深化和完善马尾新城总体规划，构建“两组团一廊道”发展格局。加快建设琅岐闽江大桥、南江滨大道东段等项目，动工建设马尾大桥、沈海高速亭江互通及接线工程等项目，抓紧东部快速通道、三江口—营前桥（隧）工程、福马铁路改建城市轻轨等项目前期工作。积极打造快安商贸文体中心、马江文化休闲综合体、江滨总部聚集区，合理布局和建设三甲医院、知名中小学校等社会事业及公共服务项目，进一步提升马尾新城综合服务功能。

　　加快提升中心城区功能品位。切实维护规划的权威性和严肃性，提高建设的水平。认真抓好海峡国际会展中心、奥林匹克体育中心、火车南站、火车北站周边及横屿、光明港片区等城市组团建设与景观改造，继续提升金山、五四北等片区，打造具有现代风貌的城市建设精品。坚持多拆少建，疏解老城区人口，实施中心城区200万平方米危旧房（棚屋区）改造，启动南街、津泰路改造，加快“城中村”改造。持续推进光明港、新店溪、新西河、潘墩河等内河综合整治，力争启动内河生态补水工程，努力实现中心城区内河“水清、河畅、路通、景美”，推进城市“绿道”、慢行系统建设。认真实施“点”、“线”、“面”攻坚计划试点，进一步改变城市环境和面貌。规范和提升住宅小区物业管理，加快解决无物业管理小区问题。持续开展市容环境卫生、交通秩序、违法建设、非法采运砂等专项整治，建设数字城管系统，提高城市综合管理、动态管理、精细化管理水平。

　　切实增强中心城市承载能力。进一步拓展对外联接通道，支持航空公司在榕设立基地公司、开辟重点航线，加快建设向莆铁路、合福铁路福州段，江阴港、可门港铁路支线，福永高速公路等项目，动工建设京台建闽高速公路、湄重高速公路福州段等项目，推进绕城高速公路东南段、福银高速公路闽清梅溪互通等一批项目前期工作。进一步完善和提升市区路网，动工建设湖东东路、浦党路等一批项目，改造提升五一南路等8条市政道路，启动实施金鸡山隧道整治等项目，争取建成螺洲大桥等12个项目。优先发展公共交通，新增更新公交车450辆，新建公交场站10个，新辟、优化公交线路47条，推进城区地下、立体公共停车场建设。全线推进地铁1号线建设，争取动工建设地铁2号线。实施地下综合管沟建设试点和交通信号灯、支路街巷路灯更新改造，力争完成市区架空缆线下地工作。加快建设福清核电站1号～4号机组等一批能源项目。推进三江口防洪工程、琅岐防洪防潮工程等防灾减灾工程建设。

　　 积极支持和融入平潭开放开发。坚持资源共享、合作共建、互利共赢，认真落实与平潭综合实验区合作框架协议。推动基础设施、市政设施等向平潭延伸拓展，支持平潭第二通道、岛外调水、供电走廊等工程建设，加快渔平高速公路延伸线、长平高速公路等项目建设。强化产业对接合作，大力争取平潭综合实验区优惠政策向福清、长乐等周边地区辐射。以“海峡号”高速客轮开通为契机，联手开发“福州—平潭—台湾”一程多站式旅游产品，构建海峡两岸海上便捷通道。继续在科技、教育、医疗卫生、人才和干部培训交流以及社会保障、公共服务等方面，为平潭综合实验区提供支持与服务。

　　 统筹推进城镇化和新农村建设。加强对县域产业发展的统筹规划，支持县（市）推进产业集聚、延伸产业链条、提升产业层次，因地制宜发展壮大县域特色经济。积极实施“大城关”、“大县城”战略，推进县(市)城关扩容提质。深化强镇扩权改革，加快省级试点小城镇、市级示范性小城镇建设，培育一批工业强镇、商贸重镇、旅游旺镇和历史文化名镇。组织开展新农村建设“百村竞赛”活动，认真实施农村“家园清洁行动”和生态家园富民工程，实现村庄规划全覆盖，打造一批综合示范精品村。持续改善农村生产生活条件，完成行政村电网改造，新建和改造农村公路200公里，新增农村客车100辆，新建农村户用沼气池2000口，新建改建农村卫生户厕5000户。加强水利建设，认真实施防灾减灾、农田水利、骨干水利、农村饮水安全、水生态环境治理等工程，新解决30万农村人口饮水安全问题，加快推进大樟溪（闽江）引调水工程、罗源霍口水库、闽清葫芦门水库等一批重大项目前期工作。加强农村“六大员”队伍建设，完善农村社会化服务体系。提高扶贫标准，继续实施整村推进扶贫开发，稳定解决扶贫对象温饱问题，实施新一轮“造福工程”搬迁3000人。加大县级基本财力保障力度，完善对口帮扶、山海协作机制，扶持财政困难县和老少边贫岛地区加快发展。

　　（四）进一步深化改革开放，精心打造良好的投资发展环境。

　　 构建更加有利于科学发展的体制机制。深化行政审批制度改革，依法最大限度减少审批事项、简化审批环节。有效盘活和统筹用好国有存量资产，做大做强市属投融资平台和产业集团，提高国有资本使用效益。拓展民间投资渠道，鼓励民营企业转型升级，支持民营经济发展壮大。大力推行政府购买公共服务，深化污水处理、垃圾处理、市容环卫、绿化养护等领域的市场化经营、产业化运作。分类推进事业单位改革。推进财政管理体制改革和统计制度方法改革。深化农村综合改革，探索建立统筹城乡一体化发展的体制机制。实施集体土地征收制度改革，提高农民在土地增值收益中的分配比例。

　　实施更加积极的大开放战略。组建招商投资促进机构，大力拓展“三维”对接，深入实施“回归工程”。加大高端制造业、高新技术产业等领域引资力度，拓展服务贸易领域的利用外资，支持上市企业返程投资。继续实施鼓励外贸发展的政策，大力开拓自贸区市场和新兴市场，推进国家科技兴贸出口创新基地和电子信息产品、船舶、家具及装饰品等重点出口商品基地建设。鼓励扩大重要资源、原材料和先进技术、关键设备进口。支持优势企业“走出去”拓展市场、购买资源、跨国经营。推进海交会市场化运营，提高第十四届海交会办会水平，积极参加第十六届投洽会等重大经贸活动。进一步凝聚侨心、维护侨益、发挥侨力。深化拓展榕港榕澳合作交流，提升海内外友城合作层次和水平。加强与长三角、泛珠三角、海西城市群及闽浙赣皖协作市的交流合作。继续做好对口支援新疆、西藏等工作。

　　拓展更加常态化的榕台交流合作。加强榕台产业深度对接，争取台商投资区扩区，争取海峡两岸汽车整车贸易口岸获批，争取设立涉台商品原产地认证办公室，加快打造海峡两岸区域性金融服务中心和大陆对台贸易中心。大力发展“海峡旅游”，力争成为赴台个人游试点城市，争取开通黄岐环马祖澳旅游线路。拓展榕台空中直航、海上直航，加快建设海峡邮政枢纽中心、邮政电讯直通口岸。深化榕台农业、科技、文化、教育、卫生、体育、民族宗教、红十字等领域的全方位交流合作，推进闽台文化创意产业园建设，办好“两马”同春闹元宵、海峡两岸合唱节、海峡版权（创意）产业精品博览交易会、海峡（福州）渔业周暨渔博会等特色对台交流活动。

　　营造更加良好的投资发展软环境。强化“四个服务”理念，办好行政服务中心，推广“厦航式”优质服务经验，切实为投资者提供主动、热情、高效、全程服务。大力推行项目代办制，开辟“企业直通车”和“项目绿色通道”。进一步推进口岸“大通关”，争取实现罗源湾港区口岸开放。切实整顿和规范市场经济秩序，着力打造“诚信福州”。坚持培养与引进相结合，不求所有但求所用，积极培养引进经济社会发展急需的高层次、高技能、高素质人才，加快建设人才公寓、国际学校和留学人员创业园、大学生创业园，营造更加良好的引才、聚才、育才、用才环境。（五）进一步强化环境保护与生态建设，积极创建国家生态市。

　　大力推进节能减排攻坚。积极推广运用节能环保新技术、新设备和新产品，推进50家工业企业重点节能项目建设，加快建陶等重点行业清洁能源改造。认真落实建设项目节能评估审查和环境影响评价、环保“三同时”等制度，全面淘汰皮革、造纸、印染、钢铁、水泥等行业落后产能，坚决取缔“十五小”、“新五小”企业。完善红庙岭垃圾处理设施，争取动工建设第二生活垃圾焚烧发电厂，加快建设洋里污水处理厂三期工程及连坂、浮村污水处理厂厂外管网，进一步提高污水集中处理率和垃圾无害化处理率。大力发展绿色经济、循环经济，加强重点企业和产业园区循环经济改造，推进可再生能源建筑应用城市示范工作与垃圾分类处置试点工作。

　　认真治理环境重点问题。全面落实环境保护“一岗双责”，加强环境执法监管，抓好创模复查工作。综合整治“三江三溪”等重点流域水环境，严控严管干流及重要支流水产网箱养殖，坚决治理畜禽养殖、石板材加工污染，保障水环境安全。深入开展环保专项行动，加强重金属、餐饮油烟、生活噪声、建筑工地粉尘、机动车尾气等污染专项治理。加强重点污染源在线监测监控，提高环境安全突发事件应对处置能力。

　　不断提升生态建设水平。认真实施“四绿”工程，造林36.5万亩。启动西湖公园榭坪屿改造，扩建晋安河、光明港沿线滨水带状公园及秀峰路沿线带状公园，实施三环一期、东二环等一批道路和重要交通路口绿化景观改造提升。加强生态公益林、沿海防护林建设管理，继续认真治理“青山挂白”、水土流失。滚动实施农村环境连片综合整治，创建50个省级以上生态乡镇、500个市级以上生态村。完善生态补偿机制，加大饮用水源保护区、自然保护区、生态功能区和闽江口湿地及生物多样性保护力度。

　　（六）进一步推动文化大发展大繁荣，加快建设文化强市。

　　 全面推进社会主义核心价值体系建设。加强理想信念和社会公德、职业道德、家庭美德、个人品德教育，大力倡导和弘扬“爱国爱乡、海纳百川、乐善好施、敢拼会赢”的福建精神和“海纳百川、有容乃大”的福州城市精神。发展健康向上的网络文化。加强未成年人思想道德建设。做好科学知识普及、人文关怀、心理疏导等工作。坚持“八个持续”、“八个提升”，构建文明创建长效机制，让文明福州持续文明。

　　大力弘扬闽都优秀文化。积极整合闽都文化资源，挖掘民俗文化、宗教文化、少数民族文化内涵，提升闽都文化知名度、影响力，争取“三坊七巷”、福建船政列入中国世界文化遗产预备名录。加大历史文化名城保护力度，抓好“三山两塔”核心区整体保护开发，启动实施朱紫坊、上下杭等历史文化街区和烟台山历史风貌区保护修复。科学合理开发永泰嵩口、长乐琴江、马尾闽安、仓山林浦和螺洲等历史文化名村（镇）资源。加强文物保护，推进非物质文化遗产、传统工艺美术的传承和创新。

　　加快提升公共文化服务水平。推进海峡图书馆、科技馆、美术馆、群艺馆及工人文化宫等项目建设，争取建成城市发展展示馆，加快打造一批高水平的公共文化设施。创新公共文化运行机制，抓好市博物馆、市歌舞剧院等公益性文化事业单位、国有文艺院团改革改制工作，鼓励社会力量兴办公益性文化事业。加大对公共文化的投入力度，推动公共文化资源向基层延伸、向农村倾斜，建设一批村级文化生态示范点、社区文化中心示范点，继续实施广播电视村村通、文化信息资源共享、县（市）数字影院建设、农村电影放映等文化惠民工程。强化文艺精品创作，在“三坊七巷”规划建设创作基地和名家工作室，积极吸引名家大师集聚。进一步繁荣文学艺术、广播影视、新闻出版和哲学社会科学，加强基层文化队伍建设，扶持发展民间文艺、群众文艺。广泛开展群众喜闻乐见的文化活动，推进中国音协福清合唱基地建设。

　　 积极培育壮大文化创意产业。加大文化产业发展扶持力度，培育壮大工艺美术、动漫游戏、文化休闲旅游、设计创意、现代传媒、文化会展、广告创意等七大重点产业。推进国家影视动漫实验园建设，争取成为海峡西岸国家级数字出版基地。提升现有文化创意产业园区发展水平，建设寿山石旅游文化城、海西创意谷、海峡文化创意产业基地、海西文化创意产业园等一批重大项目。积极打造一批大型骨干文化企业集团，培育发展新兴文化业态。

　　（七）进一步加强社会建设与管理，大力提高公共服务水平。

　　 持续推进教育优先发展。进一步优化教育资源配置，提高各级各类教育办学水平和教学质量。新建和改扩建50所公办幼儿园，大力发展普惠性、公益性幼儿园。新建和改扩建50所中小学，建设400所以上义务教育标准化学校，确保90%的中小学通过义务教育标准化评估。推进普通高中多样化发展。增强市属高校办学特色。大力发展职业教育，规范发展民办教育，办好特殊教育、老年教育、成人教育，加快构建现代化终身教育体系。深入实施素质教育，大力推进校园文化特色品牌建设，筹建学生综合实践基地。推行义务教育“小片区”管理改革，加快培养名优骨干教师。加强学校安全管理，大力推广国标校车。完善家庭困难学生资助制度。

　　切实提高全民健康水平。积极推进省立医院金山分院、利嘉医院和黄山片区医院等三甲医院建设。加快市属公立医疗机构建设发展，动工建设市二医院内科病房楼、市中医院病房楼等一批项目。改造提升18家乡镇卫生院、社区卫生服务中心和1024个村卫生所，加大基层全科医生和乡村医生培训力度。规范发展民办医疗机构。认真实施国家基本药物制度，完善药品集中采购配送机制。加强公共卫生服务，提高疾病预防控制和突发公共卫生事件应急处置能力。扶持发展中医药事业。推进居民健康信息系统平台建设，争取实现全市医疗机构就诊信息共享。规范和加强医患纠纷调处工作。筹建红十字999救援中心。深入开展爱国卫生运动。稳定低生育水平，实施“生育文明·幸福家庭”促进计划，推动人口长期均衡发展。

　　 认真筹办第八届全国城运会。把办好城运会与改善民生、提升城市有机结合起来，切实让福州因城运会而更精彩。及早备战城运会，组建成立城运会筹办机构，吸引一流人才参与策划，提高城运会组织水平。高效优质抓好海峡奥林匹克体育中心、运动员村等场馆和配套设施建设。加大优秀运动员培养引进力度，提升竞技体育水平。认真贯彻《全民健身条例》，组织开展“全民健身与城运同行”活动，争创全国全民健身示范城市。

　　大力加强和创新社会管理。加强基层基础工作，完善社区办公场所、服务设施，进一步提高村（社区）“两委”成员待遇。加大流动人口服务管理和特殊人群教育帮扶力度，实施流动人口居住证服务管理制度，建立全市流动人口综合信息服务管理平台，推进社区矫正、青少年事务社工等工作。继续办好便民呼叫中心12345系统、“中国福州”门户网站、市民卡系统、政风行风热线等便民利民公共服务平台。完善社会稳定风险评估和领导干部下访、接访、约访等制度，加强“大调解”工作，切实排查化解社会矛盾纠纷。完善社会治安防控体系，实施视频监控系统续建工程、社会视频监控资源整合工程，严厉打击违法犯罪活动，进一步提高人民群众对社会治安的满意率。全面开展企业安全生产标准化建设和创建安全社区等活动，完善公共安全预防预警和应急处置体系，有效防范和坚决遏制重特大安全生产事故发生。做好“六五”普法和法律援助、法律服务、司法鉴定等工作。进一步扩大基层民主和群众自治，支持做好村级组织换届工作。加强民族宗教工作。深入开展创建双拥模范城（县）活动，积极支持国防和部队建设，加强国防动员、民兵预备役和海防、人防、反走私等工作。

　　（八）进一步以人为本改善民生，不断提升人民群众的幸福感和满意度。

　　努力促进城乡居民收入持续增长。实施更加积极的就业政策，争取实现城镇新增就业13.3万人，转移农业富余劳动力4.6万人。加快构建城乡统一的就业援助、就业服务体系，加强职业技能培训，重点帮扶高校毕业生、农民工、就业困难人员、零就业家庭、退役军人等群体就业。支持自主创业、自谋职业、灵活就业，着力增加居民财产性和经营性收入。稳步提高最低工资标准和企业工资指导线，完善工资正常增长机制和支付保障机制。深化和谐企业创建，推行工资集体协商，加强劳动关系三方协调，维护劳动者和企业合法权益。

　　 稳步提高社会保障水平。加快完善均等化的社会保障公共服务体系，着力构建城乡一体化的社会保障工作格局，进一步扩大社会保障覆盖面。提高企业职工基本养老金，推进城镇居民养老保险制度全覆盖，继续做好新型农村社会养老保险和被征地农民养老保障工作。健全城镇职工、城镇居民基本医疗保险和新型农村合作医疗保险制度，对城乡困难居民实施大病医疗救助。完善城乡低保制度，提高城乡低保、农村“五保”供养标准和城乡社会救助水平。改善优抚对象和革命“五老”人员待遇。加快发展老龄服务事业、服务产业，力争实现城市社区居家养老服务中心、服务站全覆盖，推行互助型农村养老服务模式，加强县级社会福利中心、农村敬老院建设。强化妇女儿童、残疾人、青少年等工作，推进海峡妇女儿童活动中心、残疾人康复就业培训中心建设。加快建设救灾物资储备库。大力发展社会福利、社会优抚和社会慈善事业。

　　着力解决民生热点问题。认真实施以公共租赁房、廉租房、政策性安置房为重点的保障性安居工程建设，争取建成或基本建成2万套保障性住房，新开工建设4万套保障性住房。满足群众多层次、多样化的住房需求，促进房价合理回归，推动房地产市场健康发展。全面推广和谐征迁工作法，切实保障群众合法权益，妥善安排被征地拆迁群众生产生活出路。争取全面解决历史遗留的留用地补偿、“两权证”办理等问题。着力保障市场供应量足、质优、价稳，新增蔬菜基地1万亩，提升生猪基地31家、禽蛋基地22家，加强粮食、蔬菜、副食品储备和农副产品、主要商品价格监测预警，继续实施物价上涨与社会救助和保障标准挂钩联动、主要农副产品价格协商等机制。进一步建设“食品放心工程”，坚决治理“餐桌污染”，健全完善食品安全信息可追溯体系和监测公示制度，确保食品药品安全。坚决打击商业欺诈、制假售假行为，加强消费者维权工作，保障广大群众放心消费、安全消费。　　三、全面提高新形势下政府行政能力与服务水平

　　各位代表，在更高起点上推进福州科学发展新跨越，迫切要求我们进一步提高服务型政府建设水平。市人民政府将紧紧围绕市委提出的敢为、能为、有为的“三为”重要要求，加强和改进政府作风，提高执行力和公信力，更好地为全市人民服务、对全市人民负责。

　　（一）切实增强群众观念，提高为民服务能力。把群众呼声作为第一信号，时刻把群众放在心上，多做察民情、知民意、暖民心的工作。把群众利益作为第一选择，切实做到权为民所用、情为民所系、利为民所谋。把群众满意作为第一标准，恪尽为民之责，多做打基础、利长远、解民忧、惠民生的实事好事。

　　（二）切实增强效能观念，提高工作执行能力。大力弘扬“马上就办”的优良传统，深入开展“双争”活动，全面提升工作节奏和服务质量。大力推行“一线工作法”，少开会、开短会，少讲话、讲短话，少发文、发短文，更加及时有效地推进一线工作、解决实际问题。大力强化机关效能建设，严格绩效管理、行政问责，坚决治庸治懒，坚决反对敷衍塞责、推诿扯皮。

　　（三）切实增强学习观念，提高攻坚克难能力。倡导全员学习之风，强化学习新理论、新政策、新知识，全面打造学习型政府。建立完善先调研后决策等制度，更加积极地向基层学习、向群众学习，从人民群众当中汲取智慧和力量。注重联系实际，学以致用，切实把学习的成果转化为谋划工作的思路、推进发展的举措、化解矛盾的本领，全面提高政府工作的预见性、针对性、创造性。

　　（四）切实增强法治观念，提高依法行政能力。加强法治政府建设，严格依照法定权限和程序行使权力、履行职责。依法规范行政权力运行，强化行政执法责任，完善重大行政决策咨询、论证、听证等制度。依法接受人大及其常委会的法律监督、工作监督，主动接受政协的民主监督，广泛听取各民主党派、工商联和各人民团体、无党派人士的意见，提高人大代表议案、建议和政协提案办理质量。

　　（五）切实增强廉政观念，提高防腐拒变能力。认真贯彻廉政准则，落实党风廉政建设责任制，加强反腐倡廉教育，完善惩治和预防腐败体系，严肃查处违纪违法案件。抓好廉政风险防控工作，深化行政权力阳光运行平台建设，加强政府层级监督和行政监察、审计监督，主动接受群众监督、舆论监督。时刻牢记“两个务必”，坚决纠正损害群众利益的不正之风。

　　各位代表，站在新的历史起点，建设开放、文明、和谐、幸福的新福州，是全市人民的共同期盼，也是时代赋予我们的崇高使命。让我们紧密团结在以胡锦涛同志为总书记的党中央周围，在省委、省政府和市委的正确领导下，高举中国特色社会主义伟大旗帜，以邓小平理论和“三个代表”重要思想为指导，深入贯彻落实科学发展观，全面贯彻党的十七大和十七届三中、四中、五中、六中全会以及省第九次党代会、市第十次党代会精神，团结和依靠全市人民，进一步继往开来、凝心聚力、锐意进取、奋发图强，在更高起点上推进福州科学发展新跨越，以优异的成绩迎接中国共产党第十八次全国代表大会的胜利召开！
